# *CFTR* gene variant detection in moroccan individuals via nanopore long-read sequencing

**DOI:** 10.3389/fgene.2026.1769093

**Published:** 2026-03-24

**Authors:** Nada El Makhzen, Alexander Nater, Jean-Sébastien Rougier, Alexandre Bokhobza, Javier Sanz, Christiane Zweier, Anne-Flore Hämmerli, Rémy Bruggmann, Laila Bouguenouch, Mounia Lakhdar Idrissi, Hugues Abriel

**Affiliations:** 1 Ion Channels and Channelopathies Laboratory, Department of Medicine, Institute for Biochemistry and Molecular Medicine, University of Bern, Bern, Switzerland; 2 Department of Medicine, Graduate School for Cellular and Biomedical Sciences, University of Bern, Bern, Switzerland; 3 Department of research and innovation, Initiative Afrique of the University of Bern, Bern, Switzerland; 4 Department of Biology, Interfaculty Bioinformatics Unit (IBU) and Swiss Institute of Bioinformatics (SIB), University of Bern, Bern, Switzerland; 5 Cardiac Calcium Regulation and Nanosignalling Group, Department of Physiology, University of Bern, Bern, Switzerland; 6 Department of Human Genetics, Inselspital, Bern University Hospital, University of Bern, Bern, Switzerland; 7 Department of Medical Genetics and Oncogenetics, University Hospital Hassan II, Sidi Mohamed Ben Abdellah University, Fez, Morocco; 8 Department of Paediatrics, Hassan II University Hospital, Fez, Morocco; 9 Cluster of Research Excellence Genomics for Health in Africa, University of Bern, Bern, Switzerland

**Keywords:** CF transmembrane conductance regulator, CFTR, cystic fibrosis, long-read sequencing, oxford nanopore technology

## Abstract

**Introduction:**

Cystic fibrosis (CF) is an autosomal recessive disease resulting from pathogenic CF transmembrane conductance regulator (*CFTR*) pathogenic gene variants. While CF’s frequency varies among ethnicities, its epidemiology, clinical manifestations, and mutational profiles in Africa still must be explored due to the absence of a comprehensive public health strategy there. This study postulates that complete sequencing of *CFTR* using Oxford Nanopore Technology (ONT)-based long-read sequencing enhances the diagnostic yield.

**Methods and results:**

To amplify ∼25-kb fragments covering the whole *CFTR* gene (NM_000492.4), we designed 11 primer pairs, and barcoded libraries were prepared and sequenced on ONT flow cells (R10.4.1) using an Mk1C device. Variant pathogenicity was assessed by expressing the variant channel in HEK293 cells and examining expression through immuno-blotting. With sequencing data obtained from 9 Moroccan individuals (6 probands with suspected CF diagnoses and 3 parents), we identified the following variants: c.680T>G p.Leu227Arg, c.1521_1523del p.Phe508del, c.3484C>T p.Arg1162*, c.1090T>C p.Ser364Pro, c.3233T>C p.Phe1078Ser and c.2991G>C p.Leu997Phe. The analytical pipeline we developed allowed the phasing of the variants. Sanger sequencing confirmed all these results. The previously uncharacterised *CFTR* variants p.Ser364Pro and p.Phe1078Ser exhibit diminished expression in HEK293 cells, substantiating their pathogenic nature, with p.Phe1078Ser responding positively to the *in vitro* treatment with CFTR-modulator molecules.

**Conclusion:**

This study demonstrates the potential of long-read sequencing using ONT as an efficient means to detect CF-causing variants in African populations. Given the significant genetic heterogeneity in Africa, this technique can serve as an affordable molecular screening tool for CF, especially in areas with constrained access to genetic screening.

## 1 Introduction

Cystic fibrosis (CF) is an autosomal recessive disease caused by pathogenic variants in the ∼250-kb CF transmembrane conductance regulator (*CFTR*) gene, which affects the function of the ion channel protein to maintain chloride balance across epithelial cells. More than 2000 variants have been identified in the *CFTR* gene, with more than 700 causing CF (HGMD Professional 2023.4, CFTR2 (www.cftr2.org)), which is characterised by chronic lung disease, pancreatic insufficiency, elevated sweat chloride concentration levels, and obstructive azoospermia (Bobadilla et al., 2002). CF is estimated to affect approximately 72,000 individuals worldwide, with a prevalence of 1 in 2,000 in populations of European ancestry, a prevalence of 1 in 12,000 in South Africa’s mixed ancestry population, and an incidence of 1 in 14,000 among African American populations (El Makhzen et al., 2024). CF is a significant global health concern that affects individuals worldwide; however, many African patients continue to experience misdiagnosed CF, preventing them from receiving adequate medical care. This is mainly because many African hospitals rarely perform screening sweat tests. In an ethnically diverse population, standard 29-mutation newborn screening (NBS) kits have demonstrated a high false-negative rate of approximately 25% for children of African descent, primarily because these panels are not tailored to detect the diverse variants unique to the African continent. Despite these diagnostic challenges, clinical data suggest that the phenotypic presentations of African and Caucasian patients are comparable when they exhibit the same genetic variants (Mayer Lacrosniere et al., 2021). Molecular genetic tests necessary to diagnose CF are costly and mostly unavailable in many African countries, leading to a lack of accurate and timely molecular CF diagnosis. While the sweat chloride test remains the functional gold standard for CF diagnosis, implementing sequencing as a valuable complementary approach may be appropriate for many low- and middle-income countries, enabling comprehensive molecular characterization, improving diagnostic accuracy in atypical cases, and supporting genetic counseling (El Makhzen et al., 2024; Fuhrer et al., 2024). Furthermore, Mediterranean populations, including those of North African and Turkish origin, exhibit significant genetic heterogeneity in *CFTR* variants, common DNA variant panels designed for European populations, such as OLA, INNOLiPA, and ARMS, often show poor diagnostic sensitivity for these groups, detecting as few as 44.9% of Turkish and 69.6% of North African alleles. This high level of variant variability underscores that standard panels are insufficient for multiethnic populations, further highlighting the need for more comprehensive diagnostic tools (Lakeman et al., 2008). The time and costs associated with diagnosing CF may significantly decrease by integrating modern DNA sequencing technologies with suitable bioinformatics pipelines (Stewart and Pepper, 2017).

Developing next-generation sequencing (NGS) technologies has improved biomedical research and significantly increased the output of sequencing data. However, several studies have shown limitations that may impact its accuracy for diagnostic purposes (Chin et al., 2014; Midha et al., 2019); the short-read length (∼150 bp) is the most noticeable constraint for NGS (Midha et al., 2019). Although short-read sequencing dominated NGS until recently, there are new sequencing platforms that can produce long multi-kilobase reads. These later platforms allow for long-range haplotype creation and reference-free genomic assembly (McCombie et al., 2019). Long-read sequencing (LRS) technology may accurately characterise genetic variation and regions that are challenging to evaluate with current short-read NGS. Nevertheless, compared with short-read NGS, LRS also has limitations and challenges related to the quality of the initial material, error rates, and costs (Mantere et al., 2019). For example, DNA extraction methods and high-molecular-weight DNA handling still need improvement. In addition, raw data processing, mapping, and variant calling tools are less developed for LRS than short-read NGS (Mantere et al., 2019). Consequently, LRS has mainly been applied at this stage to look into genetic diseases with known or highly suspected disease loci (Mantere et al., 2019).

In this study, we evaluate the feasibility and efficiency of using LRS from Oxford Nanopore Technology (ONT) for complete sequencing of the ∼250-kb *CFTR* gene, encompassing both coding and non-coding regions, and to identify variants responsible for CF in African populations, initially with a focus on nine individuals from Morocco. LRS with ONT effectively identified CF-causing variants. Furthermore, we present functional evidence supporting the pathogenic classification of p.Ser364Pro, which is reported as disease-causing in CFTR-France (https://cftr.chu-montpellier.fr/cgi-bin/home.cgi?), and provide new data regarding the VUS p.Phe1078Ser identified in two Moroccan CF patients.

## 2 Materials and methods

### 2.1 Study participant recruitment and sample processing

**FIGURE 1:**
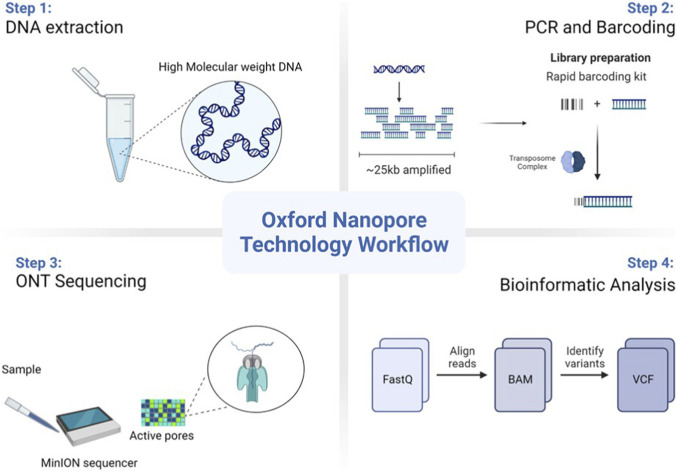
Experimental overview. Visualisation of the Oxford Nanopore Technology (ONT) sequencing workflow using the MinION device.

Fifteen individuals were referred by pediatricians from the University Hospital Hassan II (Sidi Mohamed Ben Abdellah University, Fez, Morocco) to its Laboratory of Medical Genetics and Oncogenetics. Of them, 9 were selected, including 3 healthy parents and 6 probands with a clinical suspicion of CF. This suspicion was based on various symptoms, including recurrent respiratory infections, failure to thrive, and exocrine pancreatic insufficiency. To assess the efficacy of our whole-gene sequencing approach in providing molecular clarity, we did not restrict inclusion criteria to patients with previously confirmed sweat test results. Instead, we aimed to encompass a broad spectrum of clinical presentations, ranging from classic CF cases to atypical presentations characterized by intermediate or negative sweat test results. Individual data were collected from the patient’s medical files, including each patient’s age at the onset of symptoms and upon diagnosis, as well as place of origin and presence of consanguinity. If available, we documented the *CFTR* variants and sweat test results. In addition, we assessed the results of various clinical examinations, including imaging, microbiology, and lung function tests (if they had been conducted), as well as the respiratory diseases that occurred throughout surveillance. Blood samples were then collected from those who consented to participate in the study. These samples were sent to the Medical Genetics Lab for DNA extraction and forwarded to the Ion Channels and Channelopathies Laboratory at the Institute for Biochemistry and Molecular Medicine (University of Bern, Switzerland) for LRS using ONT (Figure 1). This study was conducted in accordance with the Declaration of Helsinki and was approved by the Ethics Committee for Biomedical Research at Mohammed V University, Rabat, Morocco. Informed consent was obtained from all participants.

### 2.2 Generation of sequence data

A detailed protocol has been published on protocols. io (10.17504/protocols.io.ewov1o7y7lr2/v1).

### 2.3 Data analysis and interpretation

The bioinformatics pipeline used in this study, including basecalling, alignment, variant calling, and phasing, is publicly available at GitHub (https://github.com/alexnater/nf-ontgeno).

Unless otherwise stated, default parameters were used for all bioinformatics tools. We converted fast5 output from the sequencing to pod5 format and performed offline base-calling with Dorado v0.8.3 using the “sup” model (GitHub - nanoporetech/dorado, 2025). We performed quality control on the resulting FASTQ files using FastQC v0.12.1 and NanoPlot v1.41.6 (De Coster and Rademakers, 2023). The ONT reads were filtered with fastp v0.23.4 to remove low-quality reads (<40% of bases with base quality <15) (Chen et al., 2018) and aligned to the GRCh38 human reference genome using minimap2 v2.28 with the ‘lr:hq’ preset optimised for Q20+ ONT reads (Li, 2018). We calculated mapping and depth statistics with the SAMtools v1.20 *flagstat* command and Mosdepth v0.3.8 (Li et al., 2009; Pedersen and Quinlan, 2018). Variant calling for single-nucleotide and insertion/deletion variants (SNVs and indels) was performed for each sample with Clair3 v1.0.10 (Luo et al., 2020) using BAM files created in the mapping step. The genomic VCF (gVCF) files generated in the variant calling were then used to jointly call genotypes across the entire cohort using GLnexus v1.4.1 with the configuration file provided by Clair3 (Lin et al., 2018).

We used a two-step approach to phase the joint VCF file since the length of the overlaps between amplicons did not allow for read-based phasing across the entire *CFTR* gene. In the first step, we applied reference-based phasing with Eagle2 v2.4.1 (Lin et al., 2018) using a panel from the 1000 Genomes Project of 3,202 individuals sequenced to 30x depth (Byrska-Bishop et al., 2022). This allowed us to phase common variants found in both the reference panel and our cohort, resulting in a phasing backbone across the *CFTR* gene. To phase the rare variants not included in the reference panel, we applied a read-based phasing approach with WhatsHap v2.3 using the phased VCF output from the previous step together with the mapped ONT reads (Martin, 2016). This resulted in phasing blocks spanning the entire *CFTR* gene for all 9 individuals.

We called large structural variants (SV) from the mapped ONT reads using Sniffels2 v2.4 (Smolka et al., 2024) and cuteSV v2.1.1 (Jiang et al., 2020). For Sniffles2, we first called SV candidates in each individual separately, followed by a population-level calling step across the entire cohort using the SNF files from the first step as input. For cuteSV, we called each individual separately using the recommended settings for ONT data, followed by a merging of sample-wise VCF files with SURVIVOR v1.0.7 (Jeffares et al., 2017) (1 kb maximum breakpoint distance, minimum support of 1, accounting for SV type and strand). We then force-called the SVs in the merged VCF file in each individual using cuteSV in genotype mode and merged the resulting sample-wise VCF files again into a population-level VCF file with SURVIVOR.

Ensembl Variant Effect Predictor (VEP) (McLaren et al., 2016) was used to annotate the phased VCF files. Aligned reads were visualised using the Integrative Genomics Viewer (IGV) (Thorvaldsdóttir et al., 2012). Variants with an allele frequency >1% in public databases (gnomAD V4.0, dbSNP) were removed from further analysis. The remaining variants were prioritised based on the presence in variant databases (Clinvar, HGMD Professional 2023.4, CFTR2 (www.cftr2.org)), allele transmission (e.g., biallelic variants), and predicted variant severity. Candidate variants were predicted to be deleterious by multiple *in silico* prediction tools (Polyphen-2, SIFT). The variant classification was performed according to CFTR2 or the criteria of the American College of Medical Genetics and Genomics (ACMG) (Richards et al., 2015).

### 2.4 Sanger sequencing for the validation experiments

*CFTR* variants detected by ONT were confirmed by Sanger sequencing. The corresponding *CFTR* exons were amplified using genomic DNA as a template. They were sequenced on an ABI Prism 3500XL Genetic Analyzer (Applied Biosystems, Foster City, CA, United States) using BigDye Terminator v3.1 chemistry (Applied Biosystems, Foster City, CA, United States). Sequence data were analysed with Sequence Pilot v4.3 (JSI Medical Systems, Ettenheim, Germany).

### 2.5 Biochemical characterisation of the CFTR variants

Human embryonic kidney 293 (HEK-293) cells (ECACC, cat. #85120602) were cultured at 37 °C with 5% CO2 in DMEM medium (Gibco, Fisher Scientific, Zurich, Switzerland), supplemented with 4 mM Glutamine, 10% FBS and a cocktail of streptomycin–penicillin antibiotics. HEK-293 cells were transiently transfected with 5 µg cDNA plasmids of interest in 100-mm Petri dishes using LipoD293 (TebuBio company) *in Vitro* DNA Transfection Reagent. The cDNA plasmids of interest were p.WT-CFTR (wild-type), p.Phe508del-CFTR (a generous gift from Prof. Becq, Poitiers, France), p.Ser364Pro-CFTR, p.Leu997Phe-CFTR, and p.Phe1078Ser-CFTR (outsourced to GenScript company). Following incubation of 48 h at 37 °C with 5% CO2, cells expressing CFTR variants were washed twice with cold 1X PBS and were incubated with 4 mL of 0.5 mg/mL of EZlinkTM Sulfo-NHS-SS-Biotin (Thermo Scientific, Waltham, MA, United States) in cold 1X PBS for 30 min at 4 °C. Subsequently, the cells were washed twice with 200 mM Glycine in cold 1X PBS and once with cold 1X PBS to inactivate and remove the excess biotin, respectively. The cells were then lysed for 1 h at 4 °C with 1X lysis buffer (50 mM HEPES, 150 mM NaCl, 1.5 mM MgCl2, 1 mM EGTA (pH 8); 10% glycerol, 10% Triton X-100, 1X Complete Protease Inhibitor Cocktail). Cell lysates were centrifuged at 16,100 rcf at 4 °C for 15 min. A Bradford assay method on 590 nm was then performed using the Glomax (Promega) Plat Reader System (Switzerland) to determine the protein concentration of the supernatant. One point five mg of total protein was incubated with 50 μL Streptavidin Sepharose High-Performance beads (GE Healthcare, Uppsala, Sweden) for 2 h at 4 °C, and the remaining supernatant was kept as the input. The beads were washed four times with 1X lysis buffer before elution and incubation with 50 μL of LDS sample buffer (Invitrogen) and 100 mM DTT for 30 min at 37 °C. These biotinylated fractions were analysed as CFTR expressed at the cell surface. The input fractions, analysed as the total expression of CFTR, were resuspended with LDS Sample Buffer plus 100 mM DTT to give a concentration of 1 mg/mL and incubated at 37 °C for 30 min.

Protein samples were loaded on 8% Tris polyacrylamide gradient gels, transferred with the Trans-Blot Turbo Transfer System (Bio-Rad, Cressier, Switzerland), blocked for 1 h at room temperature with 5% milk in 1X TBS and detected with the following antibodies: 1) primary rabbit anti-hCFTR (R&D systems, cat #MAB25031), anti-α-actin (Sigma, cat #A2066) and mouse anti-α-1 Na^+^/K^+^ ATPase (Abcam ab7671), as a loading control, in a 1:1000 dilution in 1% BSA and TBS+ 0.1% Tween, and 0.02% Sodium-azide) secondary antibodies IR Dye 800 CW, anti-rabbit diluted (1:20,000) in TBS +0.1% Tween and IR Dye 700 CW, anti-mouse diluted (1:20,000). All scans were taken using the Fusion FX7 device (Witec AG, Sursee, Switzerland). Total CFTR protein expression is composed of band C, which corresponds to the mature fully-glycosylated form of CFTR (170 kDa), and band B, which corresponds to the core-glycosylated form (150 kDa); CFTR was quantified using the Fusion FX7 (Witec AG) tool and normalised to the loading control protein, α-1 Na+/K+ pump ATPase. Three independent experiments were performed. GraphPad Prism Software (version 9.5.1, La Jolla, CA, United States) was used for statistical analysis. The unpaired ANOVA was used to determine the statistical significance of the results, presented as mean ± SEM with *p* < 0.05.

### 2.6 Biotinylation assay–CFTR modulator drug treatment of the CFTR variants p.Ser364Pro, and p.Phe1078Ser

To characterize CFTR variants, p.Ser364Pro and p.Phe1078Ser, for their responsiveness to the CFTR modulator molecules—two correctors (elexacaftor and tezacaftor) and the potentiator ivacaftor—HEK293 cells were cultured and transfected following the manufacturer’s instructions described in the previous step, with the cDNA plasmids of interest. Each transfection was done twice to have two groups: a treated group with CFTR modulator drugs and a control group treated with DMSO. Six hours post-transfection, the transfected cells were treated with the drugs at the following drug concentration: elexacaftor: 8.5 µM, tezacaftor: 5 μM, and ivacaftor: 10 µM for 12 h. Biotinylation of the cells was conducted 24 h post-treatment, following the manufacturer’s instructions previously described.

## 3 Results

### 3.1 African-moroccan patients’ sequencing results

**FIGURE 2:**
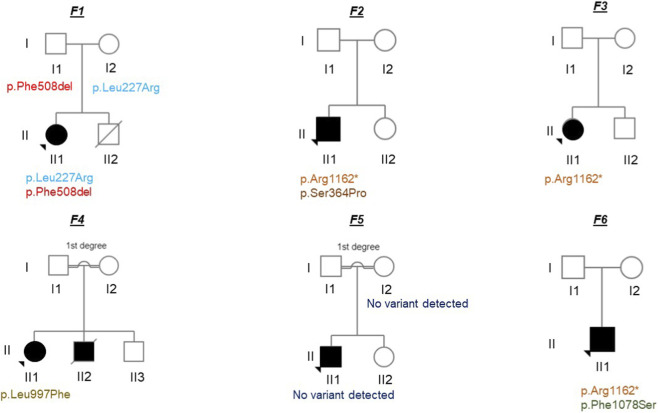
Pedigrees representing the relationship of the 9 sequenced individuals (6 probands with suspected CF and 3 parents).

We analysed the coding and noncoding regions of the complete *CFTR* gene (NM_000492.4) from 9 Moroccan-African individuals (6 probands with suspected CF diagnoses and 3 parents) using ONT amplicon sequencing (Figure 2). Sequencing metrics including per-sample yield, mapping rate, average depth, and average depth and coverage for both exons and introns were calculated using SAMtools and Mosdepth and are summarized in Supplementary Table S1.

**FIGURE 3:**
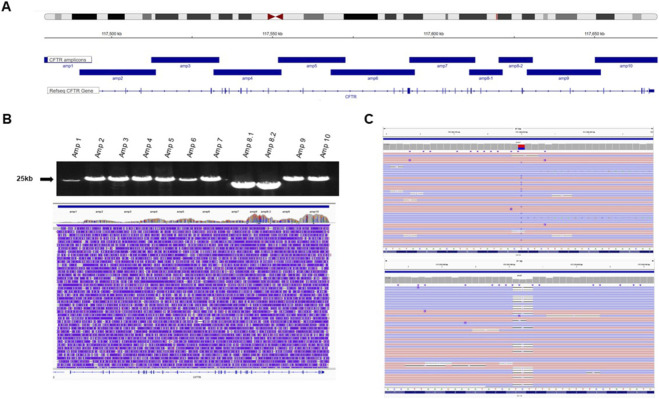
**(A)** Protocol illustration for amplifying the ∼250-kb *CFTR* gene using 11 long-range PCRs, with amp8 being split into two amplicons. **(B)** This panel demonstrates the amplification of the 11 amplicons and their subsequent sequence alignment via ONT long-read technology. **(C)** IGV visualisation of the variant *CFTR* c.1090T>C p.Ser364Pro, and *CFTR* c.1521_1523del p.Phe508del in the heterozygous state (https://igv.org/app/).

Three of the probands had positive sweat test results (chloride concentration>110 mmol/L), one had an intermediate value, one was negative, and one had no sweat test (see Table 1). Specifically, using the variant-filtering approach described in the methods section, we identified four CF-causing variants (three previously reported in the CFTR2 database and one, p.Ser364Pro, listed in the CFTR-France database), one variant of uncertain significance (VUS), and one non-CF-causing variant in the heterozygous state (Table 1). Notably, during genetic analysis, a 1-year-old CF patient (F1-II1) was identified as having two CF-causing variants, p.Phe508del and p.Leu227Arg. This patient later had a positive sweat test confirming this result, with a chloride concentration of 117 mmol/L. Furthermore, screening of the parents (F1-I1 and F1-I2) confirmed the presence of these variants and thus compound heterozygosity. The phasing efficiency was confirmed using this trio and was proven to be consistent over the majority of the *CFTR* gene, except for two apparent switch errors in (F1-I2) towards the end of the gene area (7:117,626,788–117,627,457 and 7:117,671,063–117,671,069). Additionally, a CF-causing, p.Ser364Pro (Figure 3), was identified in combination with the CF-causing variant p.Arg1162* in a patient (F2-II1) who was hospitalised at the age of 11 years with respiratory issues and exocrine pancreatic insufficiency. This patient also had a positive sweat test with a chloride concentration of 113 mmol/L. Along with the CF-causing variant p.Arg1162*, a VUS was identified, p.Phe1078Ser, in a patient (F6-II1) who was hospitalised at a young age with recurrent respiratory infections with a positive sweat test and a chloride concentration of 111 mmol/L. Due to a lack of parental samples, compound heterozygosity could not be confirmed in the latter two individuals. One proband (F3-II1) diagnosed with mild CF symptoms, along with a negative sweat test result, was identified as having one known CF-causing variant, p.Arg1162*, see Table 1, in a heterozygous state. This observation raises the possibility that this proband is a patient with a *CFTR*-related disorder. For the F4-II1 patient, only the non-CF-causing variant p.Leu997Phe was detected in a heterozygous state; this finding aligns with the individual’s negative sweat test and confirms the absence of a second pathogenic variant, despite comprehensive sequencing of the whole *CFTR* gene. For the other 2 individuals (F5-I2 and F5-II1), no pathogenic variants were detected with our sequencing. In the case of patient F5-II1, the absence of identified genetic variants is consistent with their mild clinical symptoms and intermediate sweat test results. This lack of pathogenic genetic findings underscores the clinical ambiguity associated with the intermediate chloride value, suggesting the potential for non-*CFTR*-related disorders. All the sequencing results obtained using the new ONT protocol were confirmed using traditional Sanger sequencing at the accredited genetics laboratory at the University Hospital of Bern.

**TABLE 1:** Detected genetic variants, n. p. = not performed, * Classification according to CFTR2 (www.cftr2.org), ** Classification according to ACMG guidelines *** Classification according to CFTR-France (https://cftr.chu-montpellier.fr/cgi-bin/home.cgi?). ^1^CF-causing, ^2^variant of uncertain significance, ^3^Non-CF causing.

Individual ID	Role	Allele 1	Exon/Intron	Allele 2	Exon/Intron	Sweat test
F1-II1	Proband	c.680T>G p.Leu227Arg^1,^*	Exon 6	c.1521_1523del p.Phe508del^1,^*	Exon 11	117 mmol/L
F1-I1	Parent	​	​	c.1521_1523del p.Phe508del^1,^*	Exon 11	n. p
F1-I2	Parent	c.680T>G p.Leu227Arg^1,^*	Exon 6	​	​	n. p
F2-II1	Proband	c.3484C>T p.Arg1162*^1,^*	Exon 8	c.1090T>C p.Ser364Pro^1,^ ***	Exon 22	113 mmol/L
F3-II1	Proband	c.3484C>T p.Arg1162*^1,^*	Exon 22	​	​	Negative
F4-II1	Proband	c.2991G>C p.Leu997Phe^3,^*	Exon 19	​	​	n. p
F5-II1	Proband	No variant detected	​	​	​	Intermediate value
F5-I2	Parent	No variant detected	​	​	​	n. p
F6-II1	Proband	c.3484C>T p.Arg1162*^1,^*	Exon 20	c.3233T>C p.Phe1078Ser^2,^**	Exon 22	111 mmol/L

### 3.2 Biochemical characterisation of the *CFTR* variants p.Ser364Pro, p.Leu997Phe, and p.Phe1078Ser

**FIGURE 4:**
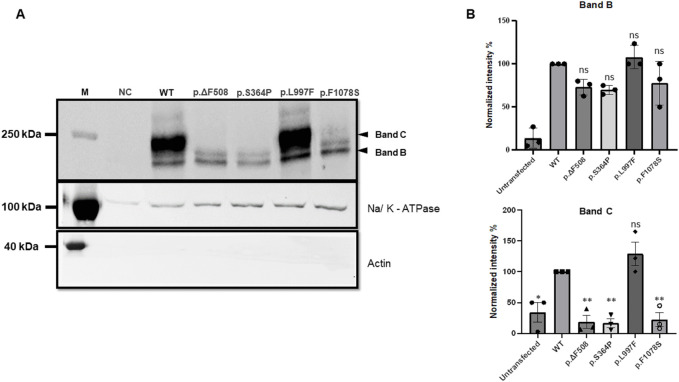
**(A)** Immuno-blots of biotinylated experiments showing the expression of p.CFTR-WT (WT) and the following 4 variants: p.Phe508del (p.ΔF508), p.Ser364Pro (p.S364P), p.Leu997Phe (p.L997F), and p.Phe1078Ser (p.F1078S). The variants p.Ser364Pro, and p.Phe1078Ser show a loss of expression that is at least as important compared with p.Phe508del. **(B)** Quantification of three different blots performed as **(A)**. Data are presented as mean ± SEM; *p < 0.05; **p < 0.01; ***p < 0.001; ****p < 0.0001; ns, non-significance, with p-value representing WT VS variant. M, Marker; NC, negative control.

To assess their possible pathogenicity, the total cellular expression of *CFTR* variants p.Ser364Pro, p.Leu997Phe, and p.Phe1078Ser were analysed, which had not been characterised previously (Table 1), after transient transfection of HEK293 cells, and their cell-surface expression by immunoblotting and cell-surface biotinylation experiments. The immuno-blotting results (Figure 4) indicate a significant decrease in whole-cell expression of the p.Ser364Pro and p.Phe1078Ser CFTR variants when compared with WT, except for the p.Leu997Phe variant, which has a similar expression as the WT. The reduced expression was similar to that observed with the positive control, the p.Phe508del variant, which is known to be significantly less expressed than its WT counterpart. These findings provide robust evidence for the pathogenicity of the p.Ser364Pro and p.Phe1078Ser variants identified in Moroccan patients. Our biochemical analyses specifically validate the disease-causing status of the p.Ser364Pro variant, listed in the CFTR-France database. Furthermore, the data indicate that p.Phe1078Ser is a likely pathogenic variant rather than of unknown significance.

### 3.3 Biotinylation assay–CFTR modulator drug treatment of the CFTR variants p.Ser364Pro, and p.Phe1078Ser

**FIGURE 5:**
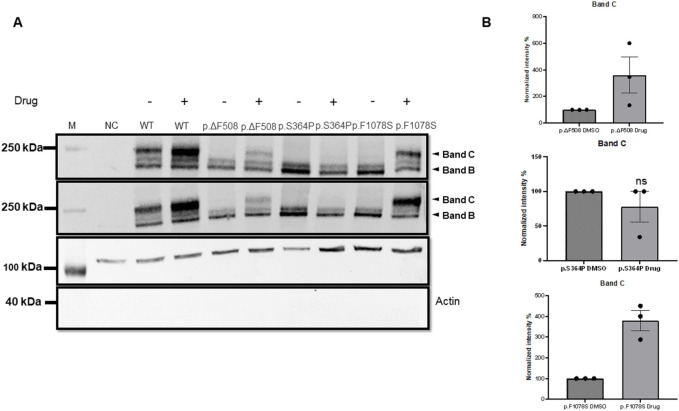
Immuno-blot of biotinylated experiments showing the expression of the wild-type CFTR (p.CFTR-WT) along with three variant forms: p.Phe508del (p.ΔF508), p.Ser364Pro (p.S364P), and p.Phe1078Ser(p.F1078S), treated (+) or not (−) was) with the Trikafta cocktail for 24 h. **(B)** Quantification of three different blots performed as **(A)**. Data are presented as mean ± SEM; *p < 0.05; ns: non-significance, with p-value representing WT VS variant. M, Marker, NC, negative control.

We then assessed the *in vitro* effects of two correctors (elexacaftor and tezacaftor) and the potentiator drug ivacaftor. After 12 h of concomitant treatment with these three molecules, we analysed the surface expression of the CFTR variants p.Ser364Pro and p.Phe1078Ser by isolating biotinylated proteins and performing immunoblot analysis. The results (Figure 5) showed a significant increase in the surface expression of the p.Phe1078Ser variant, as indicated by an increase in the intensity of the mature band C in the biotinylated fraction compared to the WT and p.Phe508del, known to be positively regulated by these compounds (Figure 5). In contrast, the p.Ser364Pro was insensitive to this treatment.

## 4 Discussion

The principal findings of this study are the following: (1) it demonstrates the use of ONT for long-read sequencing of the complete *CFTR* gene by analysing samples from 9 individuals of Moroccan-African descent. This approach efficiently covered all coding and intronic regions, demonstrating the method’s effectiveness for detailed medical genetic studies. Specifically, using this method, all 6 pathogenic *CFTR* variants in the 3 probands with positive sweat tests could be identified. (2) The accuracy of ONT sequencing was validated against traditional Sanger sequencing, with all identified variants being confirmed. (3) The analytical pipeline we developed allowed phasing of the identified variants, which is important information in recessively inherited disorders like CF. (4) Biochemical characterisation revealed a significant reduction in the expression of the p.Ser364Pro and p.Phe1078Ser variants, validating the disease-causing status of p.Ser364Pro listed in CFTR-France and indicating the potential role in CF pathology of p.Phe1078Ser variant. Notably, the p.Phe1078Ser variant showed a positive response to the CFTR modulator drug treatment.

Our complete-gene sequencing approach is comparable to that of Salakhov et al. (for the genes *MYBPC3, MYH7, TPM1, TNNT2*, and *TNNI3*), Soufi et al. (for the gene *LDLR*), and Ghukasyan et al. (for the gene *MEFV*) (Salakhov et al., 2022; Soufi et al., 2022; Ghukasyan et al., 2024). However, Soufi et al. did not aim to sequence the whole gene. Instead, they applied long-range PCR to amplify batches of exons, limiting subsequent identification of whole-gene haplotypes (Soufi et al., 2022). Similarly to our study, Ghukasyan et al. recently reported that their full-gene sequencing approach for the MEFV gene, analysing the entire gene rather than focusing solely on exons, significantly enhanced the discovery of clinically relevant variants, including those located in intronic and regulatory regions (Ghukasyan et al., 2024). Complete-gene sequencing is essential, especially for genes with known disease-causing splice variants, as specific deep intronic variants can affect splicing (de Almeida et al., 2017). Another advantage of the ONT-based method in this work is the possibility of multiplexing with manufacturer-provided standard barcodes (currently up to 96 indexes) (Frank et al., 2013). In our study, we could multiplex the long-range PCR products from all individuals into a single sequencing run. We did not try to multiplex more long-range PCR samples only because of the small number of samples.

The bioinformatics and data-processing tools available for ONT sequencing have significantly improved over the past few years. Several software tools are available for processing raw data, including Clair3 (Zheng et al., 2022). Usually, most bioinformatic protocols are included in EPI2ME Labs solutions from ONT or are recommended by them (https://labs.epi2me.io/). In our work, we developed a bioinformatic pipeline applying the Clair3 tool for calling SNVs and short indel variants. Using a combination of reference- and read-based phasing approaches, we could accurately phase the genotypes at these variants across the entire *CFTR* gene region. We also incorporated tools optimized for long-read sequencing data, Sniffles2 and cuteSV, to identify large SVs accurately. However, in the small cohort of this study, we did not detect any significant SVs. A recent study has suggested that LRS may improve the accuracy of diagnosing challenging, rare diseases by enabling the detection of SVs, repeat expansions, and other unclear gene variants (Oehler et al., 2023). In our analysis, common genetic variants were identified in individuals F4-II1 and F5-II1; however, these variants were excluded due to their exceeding the 1% allele frequency threshold in public databases, categorizing them as non-pathogenic polymorphisms. Furthermore, the application of Sniffles2 and cuteSV confirmed the absence of SVs in these patients. This rigorous filtering process and comprehensive analysis strengthen the hypothesis that their clinical presentations likely arise from non-*CFTR*-related diseases, rather than due to limitations in the sequencing methodology for detecting pathogenic variants.

For a rare disease such as CF, with limited research, especially in Africa and low- and middle-income countries, implementing LRS to diagnose CF may improve molecular diagnostic rates. We propose that this new method can be an essential complementary molecular screening tool for CF, especially in regions with limited access to genetic screening, because of the significant genetic variation in Africa. It should be noted that although a cost analysis was not part of this study, the current ONT protocol is likely more cost-effective than other NGS and long-read platforms. This affordability mainly results from the price of the sequencing device Mk1C (∼5,000 Euros) and the possibility of multiplexing the samples of many patients for whom the complete *CFTR* gene is sequenced.

Potential limitations of this study include the limited sample size, which compromises the methodology’s accuracy and applicability. To address this point, a more extensive CF-African cohort study will be initiated. The second limitation of this study is the lack of a control genotyped cohort from the Moroccan population, which will impact the assessment of the sensitivity and specificity of the current methodology.

## 5 Conclusion

In conclusion, this study provides evidence that ONT is an efficient and accurate method for identifying variants that cause CF. Our findings highlight the importance of sequencing the entire *CFTR* gene, which is especially crucial for populations with high genetic diversity, such as those found in African populations. Additionally, it underlines the need for biochemical and functional analyses to be conducted after identifying genetic variants. We propose that ONT-based sequencing represents a powerful complementary molecular diagnostic approach that can enhance variant detection and interpretation. Given its portability and scalability, this technology holds particular promise for improving access to comprehensive genetic testing in LMIC when integrated alongside established diagnostic methods.

## Data Availability

The datasets presented in this study can be found in online repositories. The names of the repository/repositories and accession number(s) can be found in the article/Supplementary Material. The sequencing data generated in this study have been deposited at the European Genome phenome Archive (EGA), which is hosted by the EBI and the CRG, under accession number EGAS50000001423. Further information about EGA can be found at https://ega-archive.org and “The European Genome-phenome Archive of human data consented for biomedical research”.
